# A New Double Digestion Ligation Mediated Suppression PCR Method for Simultaneous Bacteria DNA-Typing and Confirmation of Species: An *Acinetobacter* sp. Model

**DOI:** 10.1371/journal.pone.0115181

**Published:** 2014-12-18

**Authors:** Karolina Stojowska, Beata Krawczyk

**Affiliations:** Department of Microbiology, Faculty of Chemistry, Gdansk University of Technology, Gdansk, Poland; United States Army Medical Research Institute for Infectious Disease, United States of America

## Abstract

We have designed a new ddLMS PCR (double digestion Ligation Mediated Suppression PCR) method based on restriction site polymorphism upstream from the specific target sequence for the simultaneous identification and differentiation of bacterial strains. The ddLMS PCR combines a simple PCR used for species or genus identification and the LM PCR strategy for strain differentiation. The bacterial identification is confirmed in the form of the PCR product(s), while the length of the PCR product makes it possible to differentiate between bacterial strains. If there is a single copy of the target sequence within genomic DNA, one specific PCR product is created (simplex ddLMS PCR), whereas for multiple copies of the gene the fingerprinting patterns can be obtained (multiplex ddLMS PCR). The described ddLMS PCR method is designed for rapid and specific strain differentiation in medical and microbiological studies. In comparison to other LM PCR it has substantial advantages: enables specific species' DNA-typing without the need for pure bacterial culture selection, is not sensitive to contamination with other cells or genomic DNA, and gives univocal “band-based” results, which are easy to interpret. The utility of ddLMS PCR was shown for *Acinetobacter calcoaceticus-baumannii* (Acb) complex, the genetically closely related and phenotypically similar species and also important nosocomial pathogens, for which currently, there are no recommended methods for screening, typing and identification. In this article two models are proposed: 3′ *recA*-ddLMS PCR-*Mae*II/*Rsa*I for Acb complex interspecific typing and 5′ *rrn*-ddLMS PCR-*Hind*III/*Apa*I for *Acinetobacter baumannii* intraspecific typing. ddLMS PCR allows not only for DNA-typing but also for confirmation of species in one reaction. Also, practical guidelines for designing a diagnostic test based on ddLMS PCR for genotyping different species of bacteria are provided.

## Introduction

Bacterial strain typing, or identifying bacteria at the strain level, is particularly important for the diagnosis, treatment and epidemiological surveillance of bacterial infections. It is especially relevant in the case of bacteria exhibiting high level of antibiotic resistance or virulence as well as those involved in nosocomial or pandemic infections. Strain typing is also applicable in the study of bacterial population dynamics. Current bacterial genotyping methods are often based on the gel electrophoresis analysis of fingerprinting [1]. A particular interest can be observed in rapidly developing techniques which employ oligonucleotide adapters and are known as Ligation-Mediated PCR methods (LM PCR). These methods include, for example, AFLP (Amplified Fragment Length Polymorphism) [2]–[4]; ADSRRS fingerprinting (Amplification of DNA Surrounding Rare Restriction Sites) [5]–[7]; PCR MP (PCR Melting Profiles) [8]–[13]; and the LM PCR/Shifter method [14]. There are three major steps in the LM PCR procedure: (*i*) the restriction enzyme/s digestion of the genomic DNA and the ligation of specific adapters; (*ii*) the amplification of the restriction fragments by PCR, using a primer or primers containing common sequence of the adapter; (*iii*) the analysis of the amplified fragments using gel electrophoresis. Different mechanisms are used to select fragments of DNA for amplification. This selection depends on the physicochemical properties of nucleic acids, for example, the melting temperature of restriction fragments [8], or their length or ability to form a stable panhandle structure [5], [15]. The amplified fragments (PCR products) come from unknown regions of the genomic DNA, so the fingerprint pattern does not include any specific information about the species or genus of the tested strain. Despite the fact that these methods are characterized by a high degree of discrimination power and can be applied to a wide range of microorganisms, they require the use of the purified DNA from a single colony of correctly identified species.

Using methods typically available in today's clinical laboratories it is difficult to identify certain species of bacteria, in particular those belonging to the *Acinetobacter calcoaceticus* –*Acinetobacter baumannii* (Acb) complex, which encompasses four phenotypically and genotypically related species with valid names, i.e. *A. baumannii*, *A. nosocomialis*, *A. pittii* and *A. calcoaceticus*, and two provisional gen. sp., i.e. the so-called “Between 1 and 3” and “Close to 13TU” [16]. These six species are closely related genetically and similar phenotypically; however, they differ in their epidemiology, antibiotic resistance [17], [18], and pathogenicity [19]. Among the members of the Acb complex, clinically the most important species is *A. baumannii*. It is frequently involved in nosocomial infections, including serious outbreaks, and associated with increasing reports of multidrug-resistant strains, higher mortality rates when compared with the related species [20], [21]. More recently, non-*A. baumannii* infections, also associated to resistant strains and with different clinical outcomes [21], have been reported more often, therefore highlighting the need for accurate identification of *Acinetobacter* species. A number of genotypic methods have been proposed for *Acinetobacter* species identification [22]. The most widely used identification approaches include polymerase chain reaction (PCR) amplification and restriction analysis (PCR-RFLP/*Tsp*I) of *rec*A gene [23], sequencing of species-specific DNA regions (e.g. the intrinsic oxacillinases from different *Acinetobacter* species) [24] or, more recently, partial *rpo*B gene sequence analysis [25]. However, these methods are laborious, time-consuming, expensive, and require the use of the purified DNA from a single colony.

This paper describes a new LM PCR method called ‘double digestion Ligation Mediated Suppression PCR’ (ddLMS PCR), in which only specific restriction fragments containing a genus- or species-specific target sequence are amplified. This approach allows for strains differentiation. The restriction site polymorphism upstream from the specific target sequence leads to PCR products of varying length. It also enables identification of the species or genus of the tested strains, making it unnecessary to use genomic DNA isolated from a previously purified and identified bacterial colony. We provide a set of simplified procedures for preparing PCR templates and for characterizing the PCR product(s) directly, as well as practical guidelines for designing a diagnostic test based on the ddLMS PCR technology for genotyping different species of bacteria. We propose two diagnostic solutions: for interspecific differentiation of bacteria belonging to *Acinetobacter calcoaceticus–Acinetobacter baumannii* (Acb) complex and for intraspecific *Acinetobacter baumannii* typing.

## Materials and Methods

### Bacterial strains and DNA isolation

DNA from reference and clinical strains which were cultured from clinical samples was tested.

The reference strains of *Acinetobacter* sp. are listed in Table 1. To evaluate the ddLMS PCR method, thirty epidemiologically related and unrelated *A. baumannii* strains, previously typed and well characterized by REA-PFGE [29] and PCR MP were used. Also gram-positive and gram-negative, clinically important bacteria which do not belong to the *Acinetobacter* genus (*Escherichia coli, Klebsiella oxytoca, Klebsiella. pneumoniae, Staphylococcus aureus, Streptococcus pneumoniae, Streptococcus agalactiae, Enterococcus faecium, Enterococcus faecalis*) from the Department of Microbiology, Gdansk University of Technology, were tested. The DNA isolation from plate cultures (1–3 colonies) was performed using DNA Genomic Mini (A&A Biotechnology, Gdansk, Poland) and following the manufacturer's recommended procedure. The DNA concentration was measured using NanoDrop ND-100 (Thermo Fisher Scientific, Wilmington, USA) and ranged from 20 to 200 ng per microliter.

**Table 1:** Reference *Acinetobacter* strains use in this study.

Species/genomospecies	Source/Collection	Symbol uses in this study
*Acinetobacter baumannii*	ATCC 17978	Ab1
*Acinetobacter baumannii*	DSMZ 30007	Ab2
*Acinetobacter calcoaceticus*	DSMZ 30006	Ac1
*Acinetobacter calcoaceticus*	DSMZ 1139	Ac2
*Acinetobacter pittii*	ATCC 19004 ( = CIP 70.29)	Ap
*Acinetobacter nosocomialis*	ATCC 17903 ( = CIP 70.11)	An
*Acinetobacter gs. “Close-to 13TU”*	5804 ( = LUH 1471) SSI	C-to-13TU
*Acinetobacter gs. “Between 1 and 3”*	10095 ( = LUH 1469) SSI	1/3
*Acinetobacter junii*	DSMZ 6964	-
*Acinetobacter lwoffii*	DSMZ 2403	-
*Acinetobacter parvus*	DSMZ 16617	-
*Acinetobacter radioresistens*	DSMZ 6976	-
*Acinetobacter schindleri*	DSMZ16038	-
*Acinetobacter soli*	DSMZ 22956	-
*Acinetobacter tandoii*	DSMZ 14970	-
*Acinetobacter tjernbergiae*	DSMZ 14971	-
*Acinetobacter towneri*	DSMZ 14962	-
*Acinetobacter ursingii*	DSMZ16037	-
*Acinetobacter baylyi*	DSMZ 14959	-
*Acinetobacter beijerinckii*	DSMZ 22901	-
*Acinetobacter bouvetii*	DSMZ 14964	-
*Acinetobacter guillouiae*	DSMZ 590	-
*Acinetobacter gerneri*	DSMZ 14967	-
*Acinetobacter gyllenbergii*	DSMZ 22705	-
*Acinetobacter haemolyticus* (gs 4)	DSMZ 6962	-
*Acinetobacter venetianus*	DSMZ 23050	-

### Restriction map and oligonucleotides

Detailed restriction maps for each pair of endonucleases, *Mae*II/*Rsa*I, *Bfa*I/*Rsa*I and *Hind*III/*Apa*I were obtained by a computer analysis of the published sequence of *Acinetobacter baumannii* ATCC 17978 (GenBank Accession NC_009085). The DNA primers were designed using VNTI Invitrogen software and the oligonucleotides were synthesized and purified by IBB PAN (Warsaw, Poland).

### Detection and fingerprint pattern analysis

10 µl of the 25 µl of PCR products were analysed by agarose gel electrophoresis with ethidium bromide (2% agarose gels, 100V, for 1 h in 1x TAE buffer). Images of the gels were analysed and archived using Versa Doc Imaging System version 1000 (Bio-Rad Laboratories, Hercules, USA). The patterns obtained from the electropherograms were converted and analysed using the Quantity One software package, version 4.3.1 (Bio-Rad, San Francisco, CA, USA).The band positions in each gel were normalised using the DNA ladder. Band matching and isolate similarity was accomplished using the Dice band-based coefficient of similarity. A dendrogram was constructed using the unweighted pair group method with arithmetic averages (UPGMA).

### Model system analysis

#### Target sequence amplification (t-PCR)

The *t-*PCR was performed in 25-µl reaction volumes containing 1x buffer *Taq* with (NH_4_)_2_SO_4_ (Fermentas UAB, Vilnius, Lithuania), 2 mM MgCl_2_, 0.2 mM of each dNTP, 10 pmol of each primer (recA1 and recA2, Table 2), 1 U of *Taq* polymerase (Fermentas UAB, Vilnius, Lithuania), 1 ng of the genomic DNA. The PCRs were performed as follows: an initial denaturation at 94°C for 5 min and 25 cycles of denaturation at 94°C for 30 s, annealing at 60°C for 30 s and elongation at 72°C for 30 s. After the final cycle, the samples were incubated for 5 min at 72°C.

**Table 2:** Elements of ddLMS PCR typing systems for *Acinetobacter* sp.

ddLMS PCR typing system	Recommendation for apply	sequence of primer for *t-*PCR	Length of t-PCR	sequence of primer for *s-*PCR	Length of t-PCR
3′ *recA*-ddLMS PCR - *Mae*II/*Rsa*I simplex	INTERSPECIES TYPING - simultaneous identification and differentiation of *Acinetobacter* sp.	p-tF/recA1: 5′CTGAATCTTCTGGTAAAAC	425 bp	p-recA: GTTTTACCAGAAGATTCAGG	1 specific PCR product for each *Acinetobacter* species
		p-tR/recA2: 5′GTTTCTGGGCTGCCAAACATTAC			
5′ *rrn*-ddLMS PCR - *Hin*dIII/*Apa*I multiplex	INTRASPECIES TYPING - simultaneous identification and differentiation of *A. baumannii* strains	p-tF/16S1: 5′GGCTCAGATTGAACGCTGGCGGC	1643 bp	p-16S: TGTTAAGCCTGCCGCCAGCG	4-6^a^ different band patterns for each genetically unrelated strain of *A. baumannii*
		p-tR/16S2: 5′TACCTGTTACGACTTCACCCA			

a number of bands in fingerprint pattern could be lower than the number of copies of the target sequence within genomic DNA. That happens when PCR products generated from different part of genomic DNA (upstream from different copies of the target sequence) have the same length and it cannot be separated by gel electrophoresis.

#### Adapter preparation

The double-stranded adapter (5′ADP) for the 5′ddLMS PCR was formed by mixing 50 pmol of each adapter oligonucleotide (aLIG and aHELP*Bfa*I) with water to a final volume of 50 µl. The mixture was heated to 70°C for 2 min, and then slowly cooled to room temperature, which resulted in the formation of the adapter at a final concentration of 1 pmol/µl. The single-stranded adapter (3′ADP) for the 3′ddLMS PCR was prepared by dilution adapter oligonucleotide (aLIG*Mae*II) to a final concentration of 1 pmol/µl.

#### ddLMS PCR template preparation

25 ng of genomic DNA was digested with 5 U of the blunt-ended restriction enzyme *Rsa*I (Fermentas UAB, Vilnius, Lithuania) in 19.5 µl reaction volumes containing 1x Y/Tango restriction buffer (Fermentas UAB, Vilnius, Lithuania) for 45 min at 37°C. 5 U of the cohesive-ended restriction enzyme was then added (*Mae*II for the 3′ddLMS PCR, *Bfa*I for the 5′ddLMS PCR, Fermentas UAB, Vilnius, Lithuania). After 45 min of incubation at 65°C for *Mae*II or 37°C for *Bfa*I, the tubes were cooled at 10°C for 10 min. The master mix for the ligation contained 1 U of T4 DNA ligase (Fermentas UAB, Vilnius, Lithuania), 1x ligation buffer with ATP (Fermentas UAB, Vilnius, Lithuania), and 1 pmol adapter in each 5 µl volume. 5 µl of this ligation mixture was added to 20 µl of the digested DNAs and the tubes were incubated at 18–22°C for 1 h and then at 70°C for 10 min for the inactivation of the T4 DNA ligase.

#### Specific PCR (s-PCR)

The *s-*PCR was performed in 25-µl volumes containing 1X buffer *Taq* with (NH_4_)_2_SO_4_ (Fermentas UAB, Vilnius, Lithuania), 2 mM MgCl_2_, 0.2 mM of each dNTP, 10 pmol of the target-specific primer (p-recA, Table 2), 5 pmol of adapter-specific primer (p-ADP), 1 U of *Taq* polymerase (1 U/µl, Fermentas UAB, Vilnius, Lithuania) and 1 µl of adapter-ligated DNA fragments. The PCR was performed as follows: 5 min at 94°C to release an unligated helper oligonucleotide, 5 min at 72°C to fill in the single-stranded ends of the adapter and create amplicons, 25 cycles of denaturation at 94°C for 30 s, annealing at 60°C for 30 s and an elongation at 72°C for 90 s. After the last cycle, the samples were incubated for 10 min at 72°C. The *s-*PCR products for several reactions were sequenced to ensure that the amplification of the correct DNA target was carried out.

#### Fidelity test on the suppression PCR

The adapter-ligated DNA was used to conduct a fidelity test on a critical experimental aspect in the protocol; the formation of a proper and stable ‘panhandle’ structure, which suppresses the PCR amplification of the DNA fragments ligated with the adapter on both sides. A non-specific PCR (*ns*-PCR) was performed as described for the *s-*PCR with one exception; only the adapter-specific primer was used. The band profile of the *ns*-PCR was compared with the pattern of the *s*-PCR.

#### Primer-test PCR (pt-PCR)

The *pt-*PCR was performed to verify the target-specific primer's (p-recA) and adapter-specific primer's (p-ADP) lack of specificity to the non-digested and non-ligated DNA. The *pt-*PCR conditions were similar to those used for the *t*-PCR, with one difference; the primers were changed to adapter-specific (p-ADP) and target-specific (p-S) primers.

#### Prompt characterization of the PCR products

The *s-*PCR products of both model systems were used as templates for cross-check PCRs (*cr-*PCR) for the purpose of distinguishing the specific PCR products from false-positive ones. After agarose gel electrophoresis, the specific bands were purified from the gel using Gel-Out kit (A&A Biotechnology, Gdynia, Poland) as recommended by the manufacturer. 10 pmol of each p-crF (5′- GTGATGAACATAGAGTATTGAGG-3′) and p-crR (5′-ACCATAAATTTCGATAATACG-3′) were used for reamplification under PCR conditions similar to those used for *t*-PCR. Some *cr*-PCR products were sequenced to ensure that the amplification of the correct DNA target was carried out.

#### Test to verify the specificity of the ddLMS PCR method

Three samples were prepared, containing 25 ng of genomic DNA from (1) *A. baumannii* strain, (2) *A. baumannii* contaminated with a mixture of bacteria which do not belong to *Acinetobacter* sp. and (3) a mixture of bacteria other than *Acinetobacter* genus, respectively. The preparation of the ddLMS PCR template and the *s*-PCR for each sample were performed separately, as described above for both model systems.

### Application of the ddLMS PCR for *Acinetobacter* sp.

**Figure 1:**
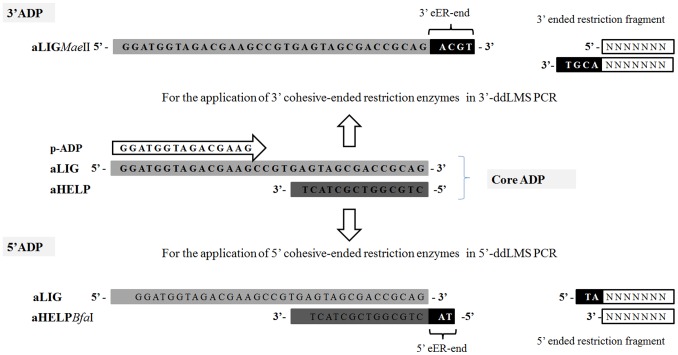
Adapters construction. Two oligonucleotides, aLIG and aHELP, comprise the core of the adapter. The cohesive-end of the adapter (eER-end) depends on the external restriction enzyme (eER) type. For the application of 5′ restriction cutters, the sequence on the 5′ end of aHELP oligonucleotide should be extended by a sequence complementary to the 5′ overhang of restriction fragment (5′ADP). For the application of 3′ restriction cutters, the 3′ end of the aLIG should be extended (3′ADP). There is no need to use aHELP oligonucleotide in 3′ADP, because the aLIG oligonucleotide acts as both ligated oligonucleotide (binding to the restriction fragment) and helper oligonucleotide (connecting the adapter to the restriction fragment by means of a complementary hybridization). The adapter-specific primer (p-ADP) sequence is shorter than the aLIG oligonucleotide and is capable of hybridizing to the 5′end of the adapter. The sequence of adapter-core oligonucleotides (aLIG and aHELP) and adapter-specific primer (p-ADP) are universal elements for all ddLMS PCR diagnostic systems.

Two types of ddLMS PCR were designed with a view to demonstrating the possibilities of the ddLMS PCR method; (1) the simplex type, 3′ *recA*-ddLMS PCR-*Mae*II/*Rsa*I which was also used as the 3′ddLMS PCR model system, for simultaneous genome-species typing and identification of *Acinetobacter* sp. based on the restriction site polymorphism upstream from *rec*A gene; and (2) the multiplex type, 5′ *rrn*-ddLMS PCR-*Hind*III/*Apa*I, for intraspecies typing of *A. baumannii* based on the restriction site polymorphism upstream from multi copy of the 16S rDNA gene. The ddLMS PCR templates, *s*-PCRs and *ns*-PCRs for 25 reference *Acinetobacter* sp. and 30 genetically related and unrelated *A. baumannii* strains of clinical origin were carried out under the conditions described for the ddLMS PCR model system. For both systems, universal elements as core-adapter (core-ADP) and adapter-specific primer (p-ADP) were used (Fig. 1). All system-specific elements such as the target-specific primer (p-S), and the pair of primers for the target sequence amplification (*t*-PCR) are presented in Table 2. *Acinetobacter* strains were previously tested by PCR-RFLP/*Tsp*I [23] and PCR MP/*Eco*RI [9].

## Results

### Outline of the ddLMS PCR method

**Figure 2:**
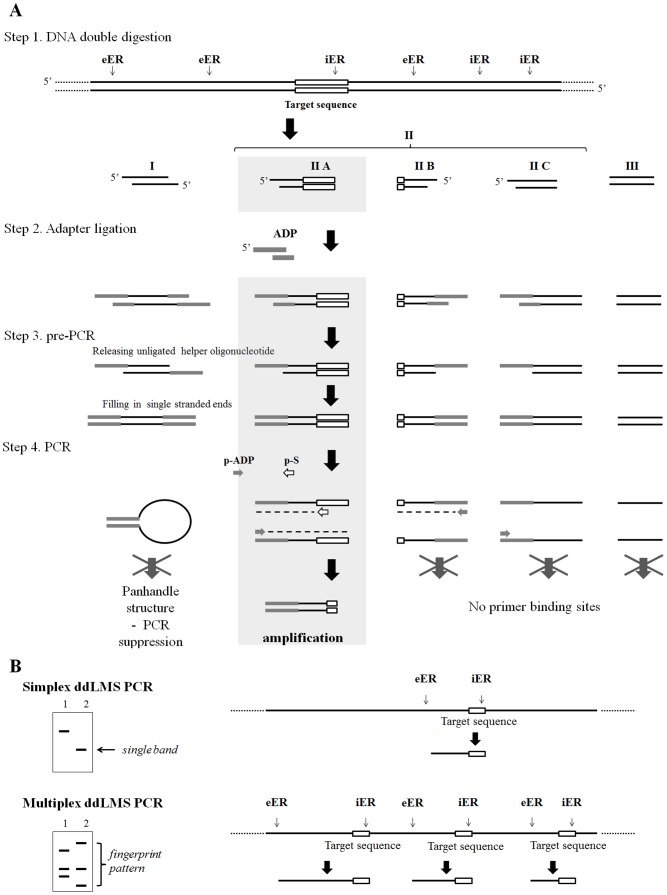
Outline of ddLMS PCR method. (A) Scheme of ddLMS PCR. eER - the external enzyme; iER - the internal enzyme; p-ADP - the adaptor-specific primer; p-S - the target-specific primer, I, II, III – sets of restriction fragments, IIA - restriction fragment digested by eER at 5′ end and iER at 3′ end, containing the target sequence with the primer p-S binding site, IIB - restriction fragment containing 3′ end of the target sequence, without primer p-S binding site, IIIB – restriction fragments without any fragment of the target sequence. (B) Two types of ddLMS PCR: simplex ddLMS PCR when there is only one copy of the target sequence or multiplex ddLMS PCR when there is more than one target sequence within genomic DNA.

The double digestion Ligation Mediated Suppression PCR method is designed to study the polymorphism of the sequence upstream from the specific target sequence (species- or genus-specific). The LMS PCR method which was used for walking in unknown genomic DNA regions from known adjacent regions was adopted with some modification to ddLMS PCR method [26]-[28]. Total DNA is digested with two restriction enzymes (Fig. 2A, step 1): the external enzyme (eER), which cuts DNA at varying distance upstream from the specific target sequence and gives varying length restriction fragments with 5‘ or 3′ overhangs, and the internal enzyme (iER), which cuts DNA within the target sequence (blunt ends are preferable). Based on ends generated by two restriction enzymes, three types of DNA fragments are formed after digestion: eEr-eER (set I), eER-iER/iER-eER (set IIA, IIB, IIC) and iER-iER (set III). The core of the ddLMS PCR is such that the target sequence is located only within some eER-iER fragments (Fig. 2A, set IIA). The mixture of DNA fragments is ligated to the synthetic ADP adapter (Fig. 2A, step 2). Two types of adapters can be used, depending on which type of eER is chosen. A double stranded adapter (5′ADP) formed by two oligonucleotides (aLIG and aHELP) is employed when the 5′ cutter is chosen, and a single stranded adapter (3′ADP) formed by one oligonucleotide (aLIG) is applied when the 3′ cutter is selected (Fig. 1). All the oligonucleotides are without phosphate group. The aHELP (for 5′ADP) or aLIG (for 3′ADP) oligonucleotides are extended by a sequence complementary to the ends produced by the external enzyme (eER-end), which allows for hybridization of the adapter to the restriction fragments. The 3′ end of the aLIG forms a permanent bond with the DNA fragment and becomes a part of the DNA template during the PCR. After ligation, all the ends produced by the eER are modified by joining the adapter, while the ends produced by the iER remain unmodified. A small amount of the DNA is used as a template for the PCR. First, the unligated oligonucleotides (aHELP) are released and the single-stranded ends are then filled by DNA polymerase (Fig. 2A, step 3). Finally, the DNA fragments are amplified by PCR using an adapter-specific primer (p-ADP), which is capable of hybridizing to the 5′ end of the core adapter, and the outwardly oriented target-specific primer (p-S), which shows a high degree of specificity to the 5′ end of the target sequence (Fig. 2A, step 4). Only restriction fragments containing the target sequence (more specifically, the primer p-S binding site) are amplified (Fig. 2A, set IIA). Fragments containing 3′ end of the target sequence (Fig. 2A, set II B) and other remaining fragments are not amplified owing to either a lack of at least one primer binding site (set IIC, III) or to their being blocked (set I). The result of the analysis in the form of the PCR products directly indicates the presence of the target sequence within the DNA tested and indirectly confirms the species or genus of the strains in question. The distance between the cutting site and the 3′ end of the target sequence is different for each strain and determined by the external enzyme (eER); the length of the PCR products thus enables differentiation of the strains among the study group. Depending on the number of copies of the target sequence within the genomic DNA, two different kinds of diagnostic assays may be designed: simplex ddLMS PCR if only one copy of the target sequence is present (Fig. 2B), or multiplex ddLMS PCR when two or more copies of the target sequence are present (Fig. 2C). The number of copies of the target sequence corresponds to the number of PCR products, which are separated by the gel electrophoresis and form a single band or the fingerprint pattern. To avoid any false-positive amplification, the phenomenon of ‘suppression PCR’ is used. In this approach, the adapter-specific primer (p-ADP) is shorter than the adapter (Fig. 1). If any DNA fragment which contains the adapter at both ends is generated (Fig. 2A, set I), then, owing to the presence of inverted terminal repeats, the ends of the individual DNA strands will form a ‘panhandle’ structure after every denaturation step. This structure is more stable than the primer-template hybrid and thus suppresses the exponential amplification.

### The model system

**Figure 3:**
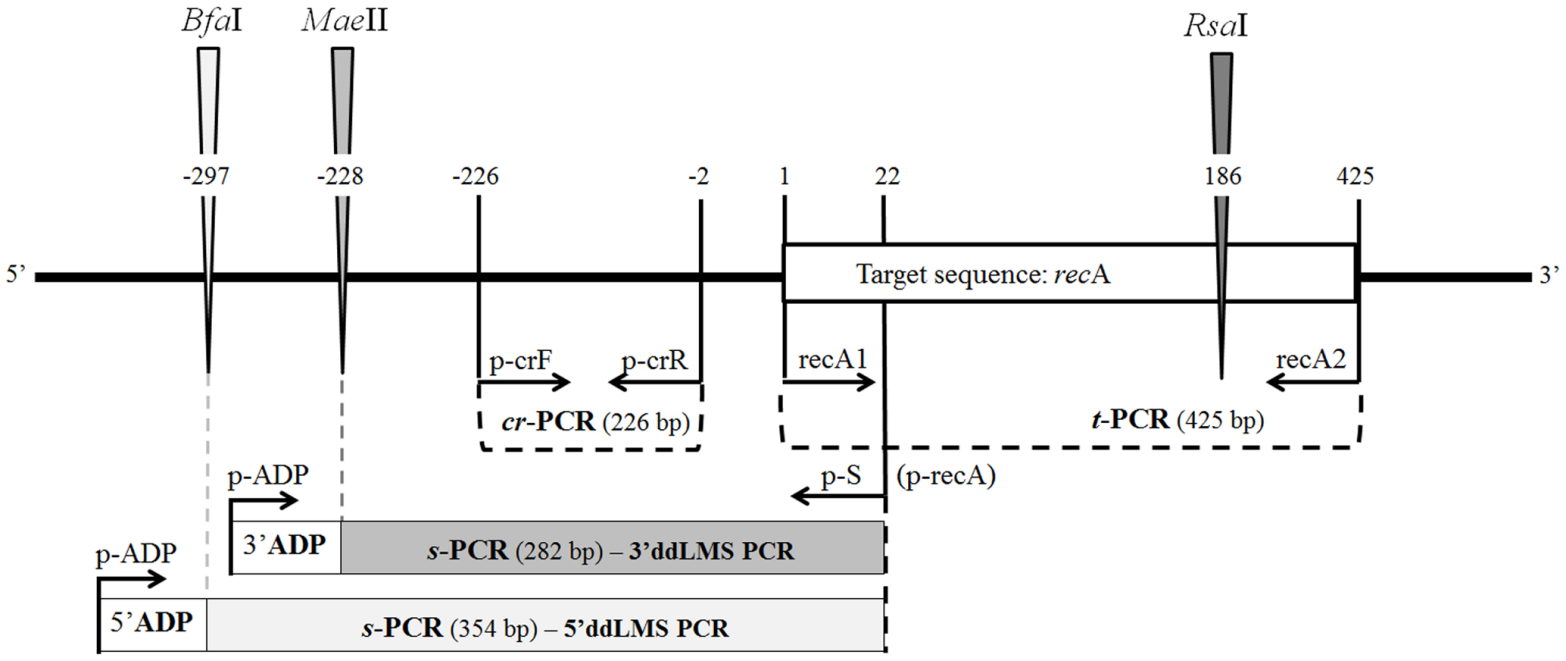
A model system of ddLMS PCR. The scheme presents a DNA region within the genomic DNA *A. baumannii* ATCC 17978 surrounding the target sequence (*rec*A gene) and the annealing position of the primers. The control PCRs such as *t*-PCR and *cr*-PCR are indicated by dashed lines; triangles indicate restriction sites for both the internal enzyme (iER – *Rsa*I) and the external enzyme (eER –*Mea*II for 3′ddLMS PCR or *Bfa*I for 5′ddLMS PCR); rectangles indicate the predicted specific PCR products for each model system. The numbers show the position (cutting side and the position of the 5′ end of the primers) in relation to the target sequence (“-” upstream, “+” downstream from the 5′ end of the target sequence); the numbers in brackets indicate the length of the PCR products. The ADP rectangle extends the s-PCRs of the adapter length.

Two model systems, a 3′ddLMS PCR and a 5′ddLMS PCR, based on the completely sequenced genome of *Acinetobacter baumannii* ATCC 17978, are shown in this study (Fig. 3). The target sequence of both models included a 425 bp region, which is located within the genomic DNA from 2,274,317 to 2,274,742 position and represents a part of the *rec*A gene. The target sequence is well-known and defined for *Acinetobacter* sp. [23]. The presence and the length of the target sequence was checked by PCR (*t*-PCR) using recA1 and recA2 primers (Table 2). The oligonucleotides used for the adapter and the adapter-specific primer (p-ADP) are illustrated in Fig. 1 and the primer directions and annealing positions in relation to the 5′ end of the target sequence are presented in Fig. 3. The length of the *s-*PCRs is calculated on the basis of the restriction maps (the length from the *Mae*II or *Bfa*I restriction site to the 5′end of the target sequence) in conjunction with the annealing positions of the adapter-specific (p-ADP) and the target-specific (p-S) primers.

**Figure 4:**
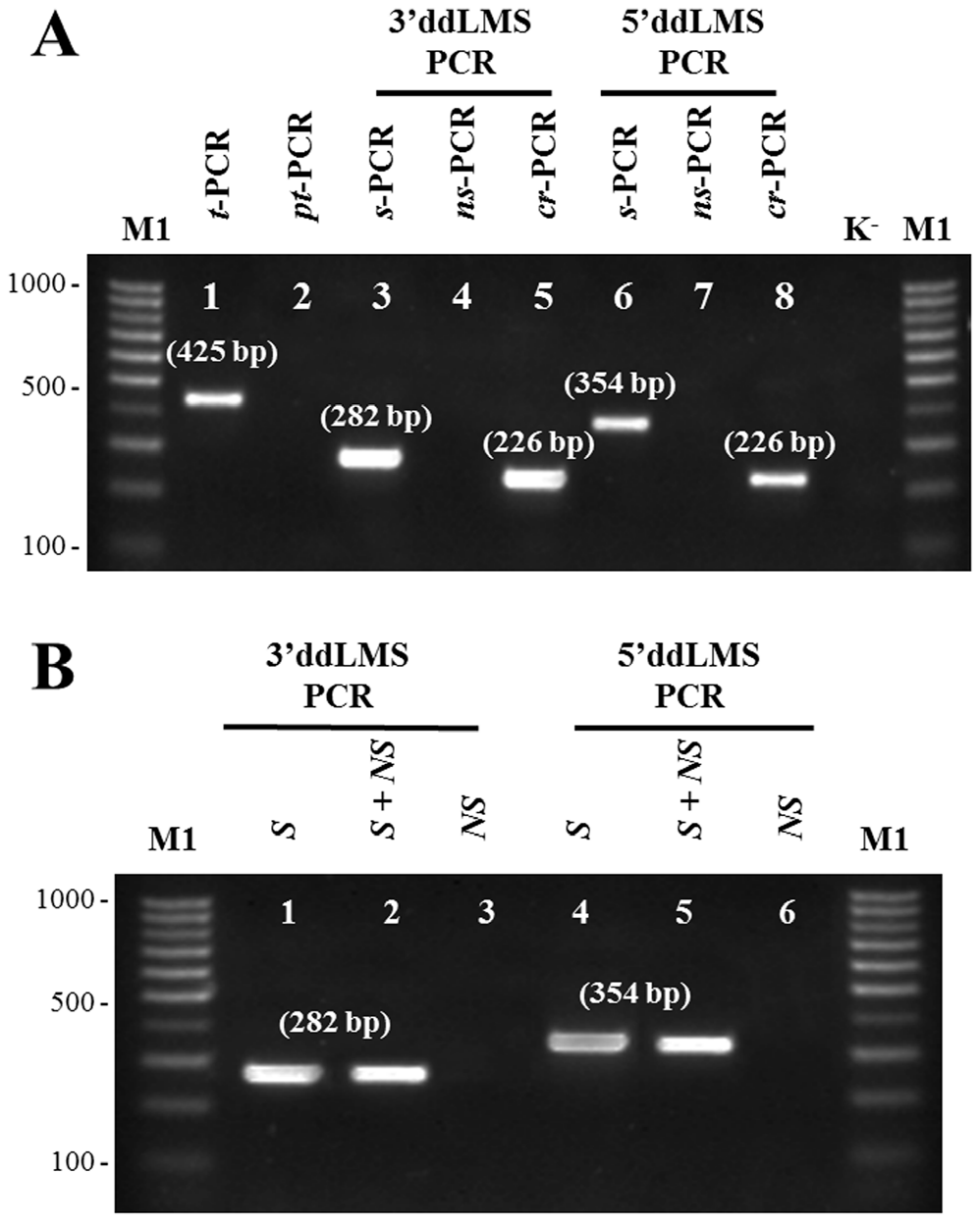
Verification results of the ddLMS PCR method. Electropherogram of PCR products: (A) model system verification, (B) test to verify the specificity of the ddLMS PCR. *t-*PCR target sequence amplification. *pt-*PCR – primer-testing PCR; *s-*PCR specific amplification, *ns-*PCR nonspecific amplification, *cr-*PCR cros-check amplification; Three different genomic DNA were used for the ddLSM PCR typing: *S*, the genomic DNA of *A. baumannii*; *S+ NS*, the genomic DNA of *A. baumannii* mixed with genomic DNA of other bacteria; and *NS*, the genomic DNA of bacteria which do not belong to the *Acinetobacter* genus. M1 the molecular DNA size marker (100–1000 bp); K^-^, the negative control of the ddLMS PCR (without DNA).

The results of the ddLMS PCR typing are clearly demonstrated by the agarose gel display, with no false positives (Fig. 4A). There is only one specific PCR product (*s-*PCR) in both the ddLMS PCR model systems (Fig. 4A, lanes 3 and 6) and the length of these *s-*PCRs corresponds to the theoretical calculation shown in Fig. 3. Three additional tests confirm that these *s-*PCRs are not false positives. The first test, *pt*-PCR (Fig. 4A, lane 2), in which we used the undigested DNA, indicates that the use of both p-S (p-recA) and p-ADP leaves no possibility of obtaining randomly amplified, unspecific PCR products from any part of the genomic DNA. The second test, *ns*-PCR, in which only p-ADP was used, indicates that in none of the restriction fragments double-digested with eER and ligated to the adapter was there any amplification which would give rise to unspecific PCR products (Fig. 4A, lanes 5 and 7). The absence of *ns-*PCRs proves that, as we had posited, these fragments form a stable ‘panhandle’ structure (see Fig. 2, set I), which effectively blocks unspecific amplification. The third test, *cr*-PCR, confirms that the *s*-PCR products are the ones expected. s-PCRs products were isolated from an agarose gel and used as a template for the cross-check PCR (*cr-*PCR). As shown in Fig. 4A (lanes 5 and 8), the *cr*-PCR leads to one product of the expected size (see Fig. 3), which represents the specific DNA fragment located upstream from the *rec*A gene in *A. baumannii* ATCC 17978 genomic DNA. All three experiments confirm that using the ddLMS PCR allows only specific DNA fragments (*s*-PCR) to be amplified, that is to say, fragments with the target sequence, which have been previously digested with a proper restriction enzyme (3′ or 5′ end cutter) and ligated to the adapter. Also, the sequencing of *s-*PCRs and *cr*-PCRs were carried out to ensure the amplification of the correct DNA target.

### The specificity of the ddLMS PCR method

The idea underlying the ddLMS PCR method is the obtaining of specific bacterial DNA from patient samples without the risk of contamination. The specificity of the diagnostic system is determined by the target-specific primer (p-S). If that is specific for only one species or genus, then other DNA from a strain of a different species/genus or contamination will not affect the ddLMS PCR results. The specificity verification test was performed in order to check these assumptions. As Fig. 4B shows, it is possible to obtain positive PCR product when the DNA contains the target sequence (Fig. 4B, lanes 1 and 2). When only the DNA isolated from bacteria which do not belong to the *Acinetobacter* genus is present in the sample (i.e., genomic DNA from other microorganisms), there is no amplification (Fig. 4B, lane 3). Moreover, the DNA contamination did not affect the efficiency and specificity of the *s*-PCR amplification (Fig. 4B, lane 2).

### Evaluation of the ddLMS PCR method

**Figure 5:**
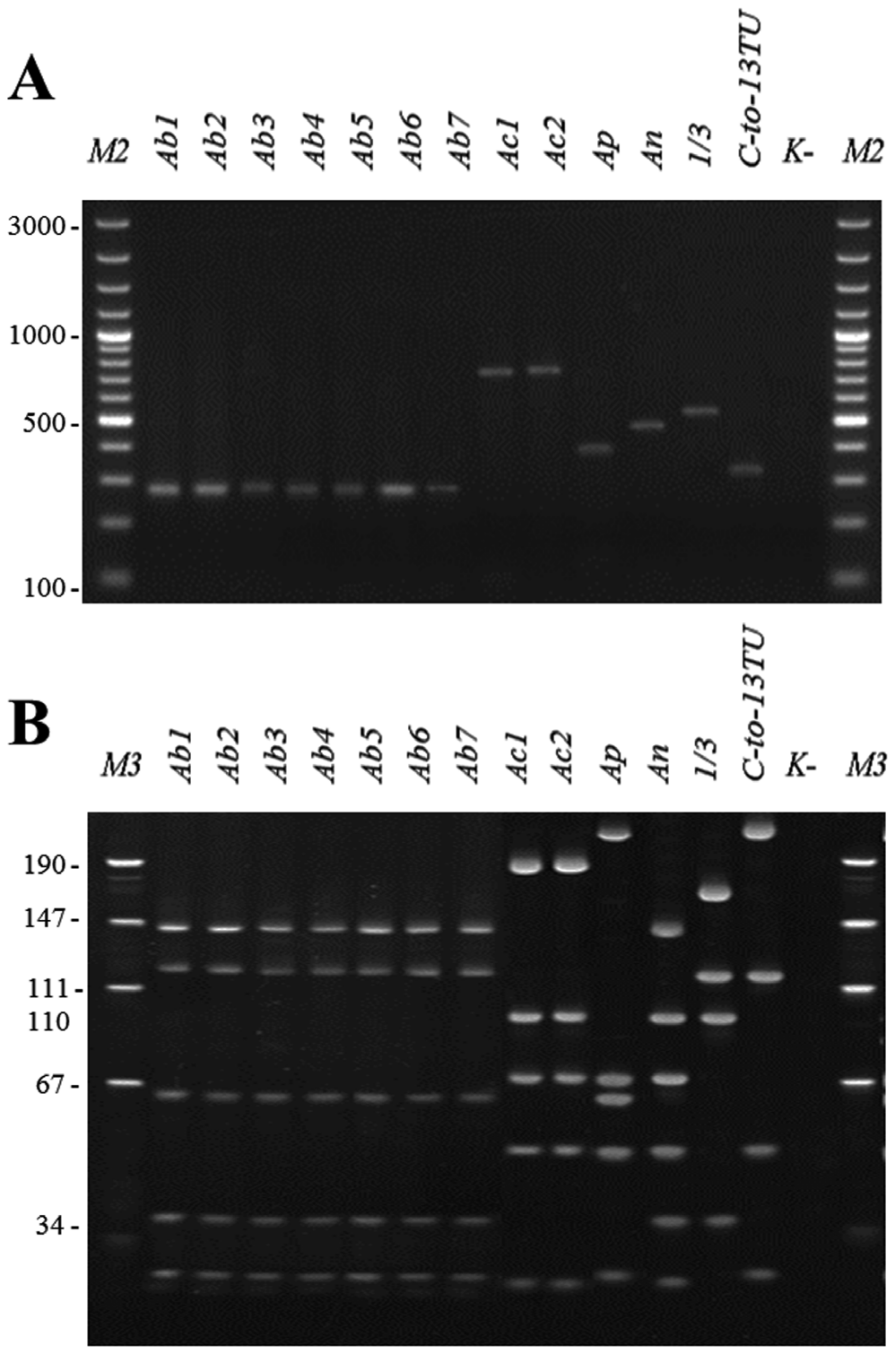
The results of Acb complex typing. (A) the 3′ *recA*-ddLMS PCR *Mae*II/*Rsa*I, (B) PCR-RFLP/*Tsp*I typing of *Acinetobacter calcoaceticus-baumannii* complex. Ab1-Ab6 – *A. baumannii* strains; Ac1-Ac2 – *A. calcoaceticus* strains; Ap – *A. pittii*; An – *A. nosocomialis*; 1/3 – *Acinetobacter* gs. “Between 1 and 3”; C-to-13TU - *Acinetobacter* gs. “Close-to 13 TU” (Table 1). M2- the molecular DNA size marker (100-3000 bp); M3 - the molecular DNA size marker (pUC19/*Msp*I); K^-^, the negative control (without DNA).

The ddLMS PCR method was designed to facilitate the simultaneous differentiation and identification of species. Within the tested group, strains are differentiated on the basis of the sequence polymorphism upstream from the target sequence. By choosing an appropriate external enzyme, these differences within the sequence can be rendered visible. In this study the 3′ *recA*-ddLMS PCR-*Mae*II/*Rsa*I system was used for interspecies typing *Acinetobacter calcoaceticus-baumannii* (Acb) complex. The sequence polymorphism upstream from the *recA* gene within *Acinetobacter* sp. is below 5% (measured by comparing the sequence in lengths of 1000 bp for the six sequenced *Acinetobacter* strains, BLAST, VNTI Invitrogene) and it only permits interspecies differentiation. We have found that *Mae*II would digest the genome sequence of *Acinetobacter sp.* at varying distances from the *rec*A gene. Typing by 3′ *recA*-ddLMS PCR-*Mae*II/*Rsa*I leads to different lengths of PCR product, depending on genomospecies: 282 bp for *A. baumannii*, 767 bp for *A. calcoaceticus,* 382 bp for *A. pitti,* 444 bp for *A. nosocomialis,* 553 bp for *Acinetobacter gs.* “Between 1 and 3” and 337 bp for *Acinetobacter* gs *“*Close to 13TU” (Fig. 5A). The differences in length of s-PCR for six species of Acb complex allow for distinguishing them by simple agarose gel electrophoresis. Identification of Acb complex species by 3′ *recA*-ddLMS PCR-*Mae*II/*Rsa*I was confirmed by PCR-RFLP/*Tsp*I according to Krawczyk et. al [23] (Fig. 5B).

The 3′ *recA*-ddLMS PCR-*Mae*II/*Rsa*I method was also tested for strains not belonging to Acb complex. There are three species for which typing by 3′ *recA*-ddLMS PCR-*Mae*II/*Rsa*I leads to s-PCR product: *A baylyi* (979 bp), *A.beijerinckii* (1690 bp), *A. haemolitycus* (620 bp) (S1A Fig.). The length of s-PCRs for these species is significantly different from Acb complex. Typing bacteria which do not belong to *Acinetobacter* sp., leads to none of s-PCR (S1B Fig.). The presence of the PCR product indicates that the strains being tested belong to the *Acinetobacter* genus, while its length indicates the particular species.

**Figure 6:**
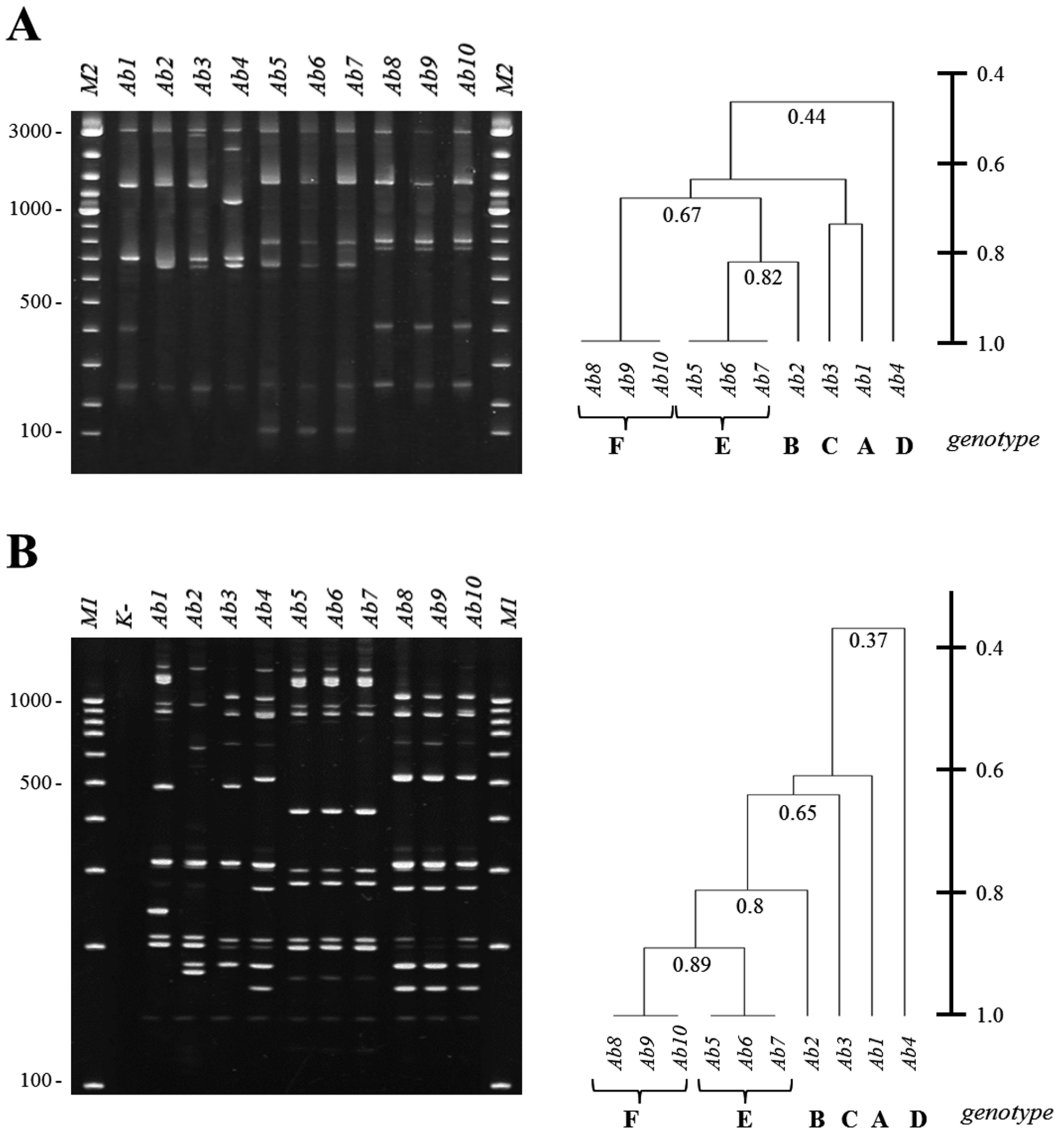
The results of *Acinetobacter baumannii* typing for representative strains. (A) 5′ *rrn*-ddLMS PCR *Hind*III/*Apa*I method and dendrogram generated by Dice Coefficient (DC) and the UPGMA clustering method, (B) PCR MP/*Eco*RI typing PCR and dendrogram generated by Dice Coefficient (DC) and the UPGMA clustering method. Ab1–Ab10 – epidemiologically related and unrelated representative *Acinetobacter baumannii* strains. A–F – genotypes, indicates different fingerprint patterns for unrelated strains. M1- the molecular DNA size marker (100–1000 bp); M2 - the molecular DNA size marker (100–3000); K^-^, the negative control (without DNA).

The discrimination level may be increased or decreased by changing the target sequence and external enzyme. If there is more than one copy of a target sequence within the genomic DNA of testing strains, a multiplex ddLMS PCR system may be designed. Each copy of the target sequence should have the same sequence and be of the same length (at least in the section between the target-specific primer hybridization site and the 5′ end of the target sequence). The level of a sequence polymorphism upstream from the target sequence should be high enough to allow the choice of an external enzyme which cuts at varying lengths from each copy of the target sequence. As a result of the analysis, several PCR products of different length, the number of which corresponds to the number of copies of the target sequence, form a fingerprint pattern. To show this, we designed the 5′ *rrn*-ddLMS PCR-*Hind*III/*Apa*I system for *A. baumannii* typing. This system is based on the sequence polymorphism upstream from the 16S rDNA gene. There are six copies of 16S rDNA within *A. baumannii*, but every copy has the same sequence and is of the same length. The target-specific primer hybridization site is specific for the *Acinetobacter* genus and is located at the 5′ end of the 16S rDNA gene. A different fingerprint pattern is shown for each unrelated strain of *A. baumannii* (Fig. 6A, genotypes A–F). Genetically related strains indicate the same fingerprint pattern (Fig. 6A, Ab5–7 as genotype E and Ab8–10 as genotype F). For other strains of *Acinetobacter* sp. which do not belong to the *A. baumannii* species, we obtained negative results (no PCR products, S2 Fig.). The presence of the PCR products indicates that strain tested belongs to the *A. baumannii* species, while the fingerprint pattern indicates the genotype of the strain. The *Acinetobacter* strain typing by 5′ *rrn*-ddLMS PCR-*Hind*III/*Apa*I was confirmed by PCR MP (Fig. 6B).

## Discussion

A new method, named ddLMS PCR, is described in this study. This method combines a simple PCR used for species or genus identification and the LM PCR methods used for strain differentiation. Strains are differentiated on the basis of a sequence polymorphism upstream from a target sequence. The target sequence (or, to be precise, the specific-primer hybridization site) is a species or genus specific sequence which affects the specificity of the diagnostic system. Within the tested group, the strains are differentiated on the basis of the polymorphism of the sequence upstream from the target sequence. The level of a sequence polymorphism affects the potential for the differentiation of strains at a particular level, but an external enzyme ultimately determines the discrimination level of the diagnostic system. A simplex ddLMS PCR or multiplex ddLMS PCR may be designed on the basis of a number of copies of the target sequence.

We used ddLMS PCR method to design a new test for both identification and typing of the Acb complex in one reaction. *Acinetobacter baumannii* which belongs to the Acb complex has emerged as a significant hospital pathogen, quickly becoming resistant to commonly prescribed antimicrobials. Detection and isolation of *A. baumannii* from surveillance and clinical cultures is laborious and requires several days due to the presence of other bacteria species present in the human flora [30]. Currently, there is no recommended medium for screening of surveillance and clinical cultures for *A. baumannii*. To identify an *A. baumannii* isolate, a couple of analyzers that could be used are Microscan WalkAway (Dade Behring, West Sacramento, CA) and Vitek 2 (bioMérieux, Marcy l′Etoile, France). However, while these analyzers will identify the isolate as *A. baumannii*, they are really only identifying the Acb complex [31]. Another method of identification is molecular testing based on rybotyping. DNA is isolated from bacterial colonies and amplified with primers selective for the 16s rDNA. The rDNA can then be sequenced and results can be compared to *Acinetobacter* genotypes base on sequence GenBank databases. This method is not useful in hospital infections (due to numerous bacterial contamination of hospital environmental and the technical problem of sequencing). The ddLMS method provides a specific tool for the detection and specific identification of *Acinetobacter baumannii* with the possibility of genetic typing. A major advantage of ddLMS-PCR, as compared to the MLST typing method, is its simplicity. The ddLMS PCR is based on only one housekeeping gene, and not based on 6–7 fragments as in the case of the MLST typing method. The method does not require sequencing, so the costs are lower. Besides, the MLST requires pure bacterial cultures, while ddLMS-PCR method is a specific PCR, not sensitive to contamination of other bacterial species. Advantages of ddLMS PCR also include simplicity in interpretation of patterns (for *recA*/*Acinetobacter baumannii* model is visible only one band) and there is no problem with the intensity of the bands as opposed to AFLP and RAPD methods.

The ddLMS PCR method is not multipurpose and requires an individual approach to each microrganism species. It is necessary to design a diagnostic system for each species or genus of microorganisms, depending on the purpose of the study and a genetic diversity of the strains to be tested. We have proposed three basic rules to follow when the design of a new system is required.

*(i) A target sequence should be chosen*. In order to design the target-specific primer (p-S), the sequence of the target gene must be known. The presence of the target sequence within the genomic DNA of the strains tested should be indicated by a simple PCR (*t*-PCR, as shown for the model systems). It is important for this sequence to be present in every tested strain.

The specificity of the ddLMS PCR method is determined by an outwardly oriented target-specific primer. The primer hybridization site should be as close as possible to the 5′ end of the target sequence. The length of the target sequence (particularly the region between the 5′ end and the target-specific primer site) should be stable for all the strains tested and for each copy of the target sequence so that it has no impact on the differences in the length of the PCR products generated by the ddLMS PCR.

When choosing the target sequence, the level of the sequence polymorphism of the preceding sequence is an important selection criterion. The level must be sufficient to differentiate strains within the tested group; this will be checked while an external enzyme is being chosen.

*(ii) An internal enzyme (iER) should be selected.* Blunt ended enzymes are preferable. If the iER generates 3′ or 5′ ends, then these ends should have a sequence different from that of the ends generated by the eER. The cutting site of the internal enzyme must be located between a target-specific primer hybridization site and the 3′ end of the target sequence. Both the presence of the location and the number of internal enzyme cutting sites can be checked by digestion of the target sequence, previously amplified by PCR (PCR/RFLP).

The internal enzyme can increase the level of specificity. The *rrn*-ddLMS PCR diagnostic system is based on the 16S rDNA gene, which is present in every strain of bacteria. The levels of the 16S rDNA gene sequence polymorphism do not allow a specific primer to be designed solely for the *A. baumannii* species. In the *rrn*-ddLMS PCR system, we were persuaded that it would be best to use the *Acinetobacter*-specific primer. In this study, the specificity of the *rrn*-ddLMS PCR system for *A. baumannii* was obtained by selecting an appropriate internal enzyme, *Apa*I, which cuts only within the 16S rDNA gene present in *A. baumannii*. For other *Acinetobacter* species, the *Apa*I cutting site is absent, which means that the restriction fragment containing the target sequence is digested with the external enzyme at both sides. Because of the inverted terminal repeats after adapter ligation, the amplification is blocked by the ‘panhandle’ structure.

*(iii) The selection of an external enzyme (eER) determines the discrimination level of the ddLMS PCR system*. For the choice of restriction enzyme, its cutting rate and % mol GC genome of tested bacteria are important. Our suggestion is to choose eER that cuts DNA with high or average frequency. The optimal length of the upstream sequence should be up to 2000 bp. Generally, if a restriction enzyme with high or average cutting frequency is used, the size of the PCR product should not be longer than 1000 bp. If the external enzyme cuts DNA at a varying distance upstream from the target sequence for each strain within one species, the ddLMS PCR system can be used for intraspecific strain differentiation. If the distance from an external enzyme cutting site to the 5′ end of the target sequence is unchanged for strains of the same species, but different for different species of the same genus, then the ddLMS PCR system can be used as a species-identifying test. The eER has to generate either 3′ or 5′ sticky ends in order to increase the efficiency and specificity of the ligation to the adapters. If the sequence of the genomic DNA or the sequence surrounding the target sequence is known, eER can be chosen theoretically, on the basis of a comparison of a sequence preceding the target sequence. Otherwise, the enzyme should be selected experimentally, based on at least three genetically unrelated strains.

There are two universal elements in the ddLMS PCR; the core ADP and adapter-specific primer (p-ADP), sequences of which are presented in Fig. 1. The eER-ends which extend the aLIG (for 3′ADP) or the aHELP (for 5′ADP) are not universal and their selection depends on the external enzyme (eER) which has been chosen.

The results produced by the ddLMS PCR are easy to interpret. A single PCR product (simplex ddLMS PCR) or a fingerprint pattern for several bands (multiplex ddLMS PCR) are characteristic of each strain (intraspecific differentiation) or species (interspecific differentiation). In order to analyze the results, a simple electrophoretic separation in agarose gel is all that is needed. In comparison to the LM PCR methods where acrylamide gels are used, the separation of PCR products and visualization takes less time. The presence of PCR product/products provides direct confirmation of the species or genus of the tested strains. No special tools or software are needed for the differentiation of strains; all that is required is a visual comparison of the electrophoretic patterns with the DNA molecular mass. The low number of bands makes the electrophoretic pattern readable.

The simplex ddLMS PCR is a binary method. Based on the results, the strains being tested can only be assigned to a genotype or species. The length of the PCR product includes no information concerning the relationship between strains because the shift of the external-enzyme cutting site is not dependent on the degree of similarity between the strains. On the other hand, though, the simplicity of the simplex ddLMS PCR method makes it possible to replace the PCR step with a real-time PCR. The differences in the length of the PCR products can easily be translated into differences in the melting temperature.

Unlike the simplex ddLMS PCR, the multiplex ddLMS PCR method enables the measurement of the genetic similarity between the tested strains. Using simple cluster analysis methods, a dendrogram graph can be generated. As shown in Fig. 6A, typing by 5′*rrn*-ddLMS PCR-*Hind*III/*Apa*I distinguishes six genotypes in the set of ten *A. baumannii* strains (A–F). The genotypes indicate varying level of genetic similarity. Strains are grouped by PCR MP (Fig. 6B). The dendrogram analysis could be used to study genetic relationships, bacterial population dynamics, and the track and source of bacterial infection.

By simplex ddLMS PCR a mixture of several *Acinetobacter* species belong to Acb complex can be typed. In that case, the s-PCR products of different length form a band pattern in which each band represents one species. This is a great solution for diagnosing mixed infections of Acb. Without isolation and separation of different strains it gives the possibility to test how many and which species are in a specimen. This can significantly reduce the cost and duration of the analysis. However, lack of a PCR product only does not indicate, that there is no bacteria in the sample. It indicates that only *Acinetobacter* sp. (Acb) strain/s are absent. DNA of other bacteria does not produce PCR product/s.

Owing to the fact that the ddLMS PCR analysis can be carried out entirely in a closed system, the sensitivity and specificity of the method may be increased and the time of the analysis can be reduced. Thus, the ddLMS PCR method could be used not only by academic but also by medical, microbiological and diagnostic laboratories.

## Conclusions

The double digestion Ligation Mediated Suppression PCR method uses the polymorphism of the sequence preceding the target sequence for the molecular typing of microbes. The combination of a target sequence and an external restriction enzyme enables the creation of different diagnostic systems, with varying degrees of discrimination level. This method combines the specificity of a simple PCR and the ability to differentiate between the strains characteristic of LM PCR techniques. It can be used for either bacteria- or yeast-specific differentiation. The fact that the ddLMS PCR is not sensitive to contamination is a great advantage. DNA from another microorganism for which the diagnostic kit is not designed does not affect the efficiency and specificity of the specific DNA fragment amplification. Contaminated cells or contaminated genomic DNA can be used for this approach; pure genomic DNA is not required. The ddLMS PCR method is designed for rapid and specific strain differentiation in medical and microbiological studies.

## Supporting Information

S1 Fig **The results of the 3′** ***recA*** **-ddLMS PCR** ***Mae*** **II/** ***Rsa*** **I typing.** (A) *Acinetobacter* sp. not belonging to Acb complex, (B) species not belonging to *Acinetobacter* sp. M2- the molecular DNA size marker (100–3000 bp); K^-^, the negative control (without DNA).
(TIF)

S2 Fig **The results of Acb complex typing by 5′** ***rrn*** **-ddLMS PCR** ***Hind*** **III/** ***Apa*** **I.** Ab1, Ac1, etc. (Table 1), M2 - the molecular DNA size marker (100–3000); K^-^, the negative control (without DNA).
(TIF)
