# Supplementary figures and images for: A New Double Digestion Ligation Mediated Suppression PCR Method for Simultaneous Bacteria DNA-Typing and Confirmation of Species: An Acinetobacter sp. Model
Source: PLoS One. 2014 Dec 18;9(12):e115181. doi: 10.1371/journal.pone.0115181 (PMC4270756; doi:10.1371/journal.pone.0115181)

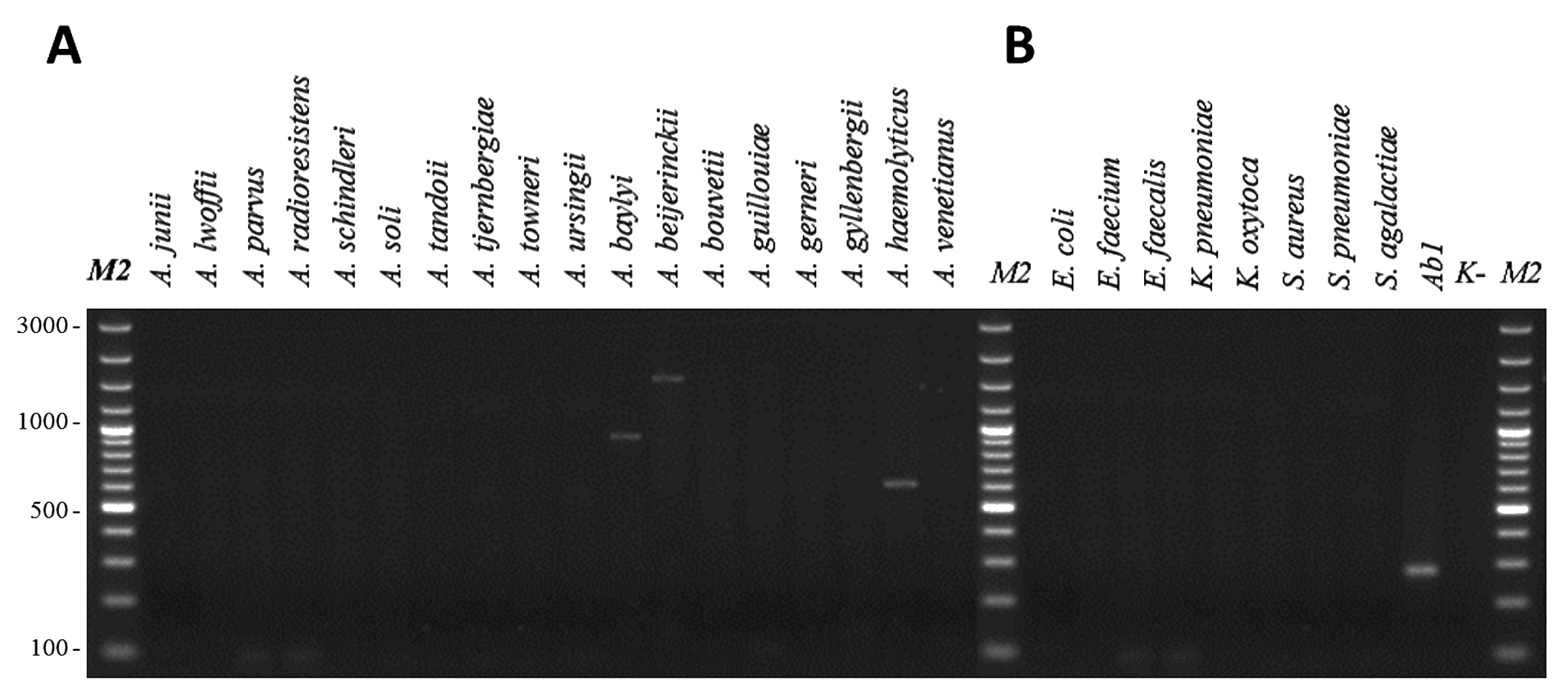

Supplement: S1 Fig — The results of the 3′ recA -ddLMS PCR Mae II/ Rsa I typing. (A) Acinetobacter sp. not belonging to Acb complex, (B) species not belonging to Acinetobacter sp. M2- the molecular DNA size marker (100–3000 bp); K-, the negative control (without DNA). (TIF) [file pone.0115181.s001.tif]

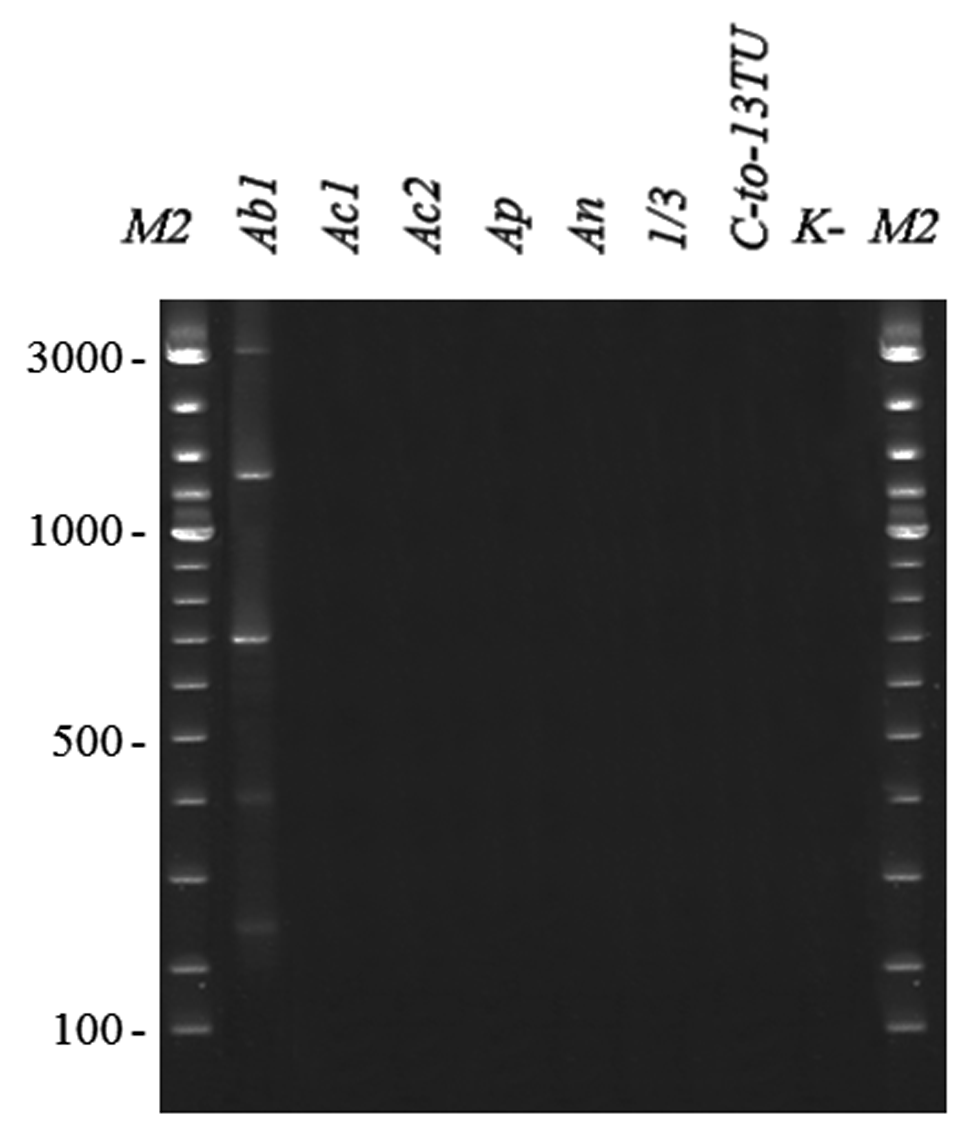

Supplement: S2 Fig — The results of Acb complex typing by 5′ rrn -ddLMS PCR Hind III/ Apa I. Ab1, Ac1, etc. (Table 1), M2 - the molecular DNA size marker (100–3000); K-, the negative control (without DNA). (TIF) [file pone.0115181.s002.tif]
